# Regulation of total LC3 levels by angiotensin II in vascular smooth muscle cells

**DOI:** 10.1111/jcmm.17215

**Published:** 2022-02-03

**Authors:** David Mondaca‐Ruff, Clara Quiroga, Ignacio Norambuena‐Soto, Jaime A. Riquelme, Alejandra San Martin, Mario Bustamante, Sergio Lavandero, Mario Chiong

**Affiliations:** ^1^ Advanced Center for Chronic Diseases (ACCDiS) Facultad Ciencias Químicas y Farmacéuticas & Facultad Medicina Universidad de Chile Santiago Chile; ^2^ Advanced Center for Chronic Diseases (ACCDiS) Facultad Medicina Pontificia Universidad Católica de Chile Santiago Chile; ^3^ Division of Cardiology Department of Medicine Emory University Atlanta Georgia USA; ^4^ Department of Internal Medicine (Cardiology Division) University of Texas Southwestern Medical Center Dallas Texas USA; ^5^ Corporacion Centro de Estudios Cientificos de las Enfermedades Cronicas (CECEC) Santiago Chile

**Keywords:** angiotensin II, AT1 receptor, autophagy, Bag3, LC3, ROCK

## Abstract

Hypertension is associated with high circulating angiotensin II (Ang II). We have reported that autophagy regulates Ang II‐induced vascular smooth muscle cell (VSMC) hypertrophy, but the mechanism mediating this effect is still unknown. Therefore, we studied how Ang II regulates LC3 levels in VSMCs and whether Bag3, a co‐chaperone known to regulate LC3 total levels, may be involved in the effects elicited by Ang II. A7r5 cell line or rat aortic smooth muscle cell (RASMC) primary culture were stimulated with Ang II 100 nM for 24 h and LC3 I, LC3 II and Bag3 protein levels were determined by Western blot. MAP1LC3B mRNA levels were assessed by RT‐qPCR. Ang II increased MAP1LC3B mRNA levels and protein levels of LC3 I, LC3 II and total LC3 (LC3 I + LC3 II). Cycloheximide, but not actinomycin D, abolished LC3 II and total LC3 increase elicited by Ang II in RASMCs. In A7r5 cells, cycloheximide prevented the Ang II‐mediated increase of LC3 I and total LC3, but not LC3 II. Moreover, Ang II increased Bag3 levels, but this increase was not observed upon co‐administration with either losartan 1 μM (AT1R antagonist) or Y‐27632 10 μM (ROCK inhibitor). These results suggest that Ang II may regulate total LC3 content through transcriptional and translational mechanisms. Moreover, Bag3 is increased in response to Ang II by a AT1R/ROCK signalling pathway. These data provide preliminary evidence suggesting that Ang II may stimulate autophagy in VSMCs by increasing total LC3 content and LC3 processing.

## 1 INTRODUCTION

Circulating angiotensin II (Ang II) levels are increased in hypertensive patients and involved in vascular damage.^1^, ^2^ Ang II binds to the angiotensin type 1 receptor (AT_1_R) in vascular smooth muscle cells (VSMC) and activates the RhoA/Rho‐associated protein kinase (ROCK) signalling.^3^

Autophagy is characterized by the formation of a double‐membrane structure named autophagosomes, where cytosolic components are sequestered and then degraded through membrane fusion with lysosomes.^4^ The formation of the autophagosome from the phagophore involves several autophagy‐related (ATG) proteins, including LC3.^4^, ^5^ Pro‐LC3 is proteolized by Atg4, generating LC3 I and then is conjugated with phosphatidylethanolamine (PE), forming LC3 II.^4^, ^5^ Given that LC3 II remains bound to the autophagosome membrane until its degradation, LC3 II protein levels are used as an autophagy marker.^4^ Exploring further mechanisms governing LC3 levels, Bag3 emerges as a likely candidate. This protein is a co‐chaperone that modulates the Hsp/Hsc70 chaperone, with an important participation in protein folding and cellular stress response.^6^ Moreover, we previously showed that Bag3 regulates total LC3 levels by modulating its mRNA translation.^6^ A total increase in LC3 levels is associated with increased basal autophagy.^6^ Also, we have reported that Ang II induces autophagy by increasing LC3 II protein levels in VSMC.^7^ We, therefore, evaluated whether Ang II also regulates total LC3 levels in VSMC and if this effect is regulated by the AT1R/ROCK signalling pathway.

## 2 MATERIALS AND METHODS

### 2.1 VSMC and primary cell culture

VSMC, A7r5 cell line (ATCC, CRL‐1444) were cultured as described.^8^ Rat aortic smooth muscle cells (RASMC) primary culture were isolated and cultured from Sprague‐Dawley rats as described.^7^

### 2.2 Western blot

A7r5 cells and RASMC were lysed using the RIPA lysis buffer. Proteins were separated in polyacrylamide gels (SDS–PAGE, 10%–15%), electrotransferred to PVDF membranes, and blocked with 5% defatted milk in 0.1% (v/v) of TBS‐Tween 20 (TBS‐T). Membranes were incubated at 4°C overnight with primary antibodies. Detection and quantification of bands were performed as described.^7^ Protein content was normalized to GAPDH or β‐tubulin.

### 2.3 mRNA extraction and RT‐qPCR

Total mRNA from RASMC were extracted using the Trizol method. cDNA was synthetized from 500 ng of total RNA. Using specific primers for MAP1LC3B, amplification was performed in StepOne Real‐Time PCR‐system (Thermo Fisher Scientific). YWHAZ was used as a housekeeping gene.^9^ After checking the amplification efficiency (95%–105%), the $\Delta \Delta C_{t}$ method was used for the analysis. MAP1LC3B mRNA levels were measured by RT‐qPCR in RASMC using the following primers: forward 5′‐CGTCCTGGACAAGACCAAGT‐3’ and reverse 5'‐CCATTCACCAGGAGGAAGAA‐3′.

### 2.4 Statistical analysis

All data are presented as mean ± standard error of the mean (SEM). One‐way ANOVA with post hoc or Student *t*‐tests were performed using Graphpad Prism 5 (Graphpad). A *p* value of *p* < 0.05 was considered as statistical significance.

## 3 RESULTS

### 3.1 Ang II stimulates autophagy in VSMC

**FIGURE 1:**
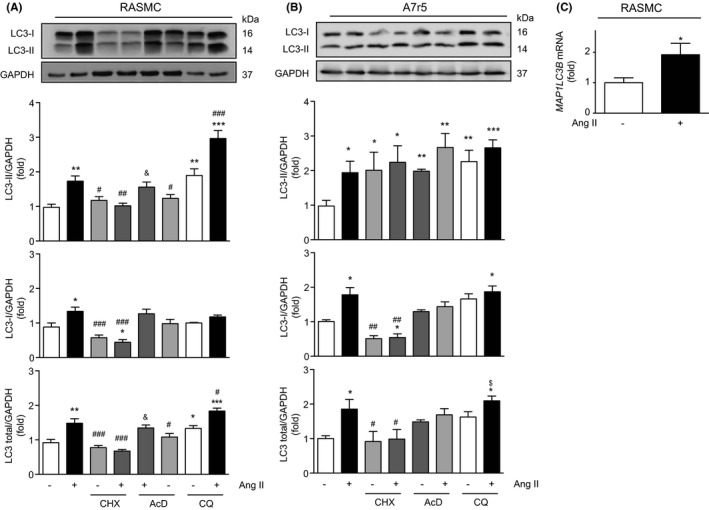
Ang II increases LC3 protein levels through transcriptional and translational mechanisms in VSMC. (A) RASMC and A7r5 cells (B) were pre‐treated with cycloheximide (CHX, 100 μM) or actinomycin D (AcD, 5 μM) 1 h before treatment with 100 nM of Ang II for 24 h. Cells were incubated with CQ (30 μM) for the last 4 h of stimulation. LC3 protein content was evaluated by Western blot. (C) RASMC were treated with Ang II for 24 h, and MAP1LC3B mRNA levels were determined by RT‐qPCR. YWHAZ was used as a loading control. Data shown as mean ± SEM. *p < 0.05, **p < 0.01, ***p < 0.001 vs control; ^#^ *p* < 0.05, ^##^ *p* < 0.01, ^###^ *p* < 0.001 v/s Ang II; ^$^ *p* < 0.05, vs CQ; ^&^ *p* < 0.05 vs AcD. Statistical analysis was performed using one way ANOVA, followed by Newman–Keuls post‐test, or Student *t*‐test (*n* = 3–5)

We previously described that Ang II induced autophagy in VSMC.^7^ However, Ang II (100 nM for 24 h) also stimulated significant increases in LC3 I and total LC3 (LC3 I + II) protein levels in RASMC and A7r5 cells (Figure 1A,B). Moreover, when autophagic flux was evaluated, the same increase in LC3 II and total LC3 protein levels were observed in RASMC (Figure 1A), whereas in A7r5 cells, LC3 I, LC3 II and total LC3 protein levels were increased (Figure 1B).

### 3.2 Ang II increased LC3 levels by transcriptional and translational mechanisms in VSMC

Given that Ang II increased total LC3 protein levels (Figure 1A,B), we sought to assess whether this peptide increased LC3 protein levels through transcriptional or translational mechanisms. The results show that MAP1LC3B mRNA was increased in RASMC after treatment with Ang II (Figure 1C). Next, we pre‐treated primary RASMC for 1 h with either actinomycin D 5 µM or cycloheximide 100 µM, inhibitors of RNA and protein synthesis respectively. Then, cells were treated with or without Ang II. Cycloheximide prevented the increase in LC3 II protein levels induced by Ang II in RASMC. The same effect was found in LC3 I and total LC3 levels (Figure 1A). However, when actinomycin D was added, the induction in LC3 II and total LC3 protein levels by Ang II was still observed, but this effect was not observed with LC3 I protein levels (Figure 1A). Furthermore, in A7r5 cells, cycloheximide prevented the Ang II‐mediated increase of LC3 I and total LC3, but not LC3 II (Figure 1B). Treatment with actinomycin D prevented the increase in LC3 I and total LC3 protein levels, but had no effect in LC3 II (Figure 1B).

### 3.3 Ang II increased Bag3 protein levels in VSMC

**FIGURE 2:**
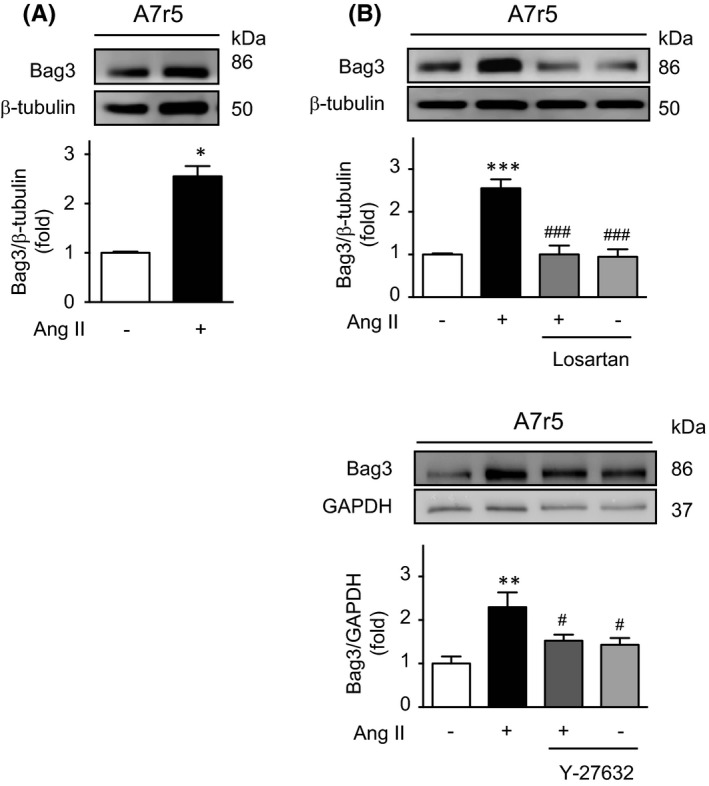
Ang II increases Bag3 in A7r5 cells. (A) A7r5 were treated with Ang II 100 nM during 24 h, and Bag3 protein content was assessed by Western blot. (B) Upper panel: A7r5 cells were pre‐treated with losartan 1 μM for 1 h before incubation with Ang II 100 nM for 24 h, and protein content of Bag3 was evaluated by Western blot. Lower panel: A7r5 were pre‐treated with Y‐27632 10 μM, 1 h before stimulation with Ang 100 nM for 24 h. Bag3 protein content was assessed by Western blot. β‐Tubulin served as loading control. Protein levels of all experiments are normalized by β‐tubulin. The data are shown as mean ± SEM, *n* = 3–5. *p < 0.05, **p < 0.01, ***p < 0.001 vs control; ^#^ *p* < 0.05, ^###^ *p* < 0.001 vs Ang II. Statistical analysis was performed using Student *t*‐test, or one way ANOVA, followed by Newman–Keuls post‐test (*n* = 3–5)

Given that Bag3 can regulate autophagy by controlling total LC3 protein levels by a translational mechanism,^6^ we explored whether Ang II can modulate the protein levels of this co‐chaperone. Ang II significantly increased Bag3 protein levels (Figure 2A), but this effect was prevented by AT1R antagonism with losartan 1 μM (Figure 2B) and ROCK inhibition with Y‐27632 10 μM (Figure 2B).

## 4 DISCUSSION

Our present study shows that Ang II regulates LC3 protein levels by transcriptional and translational mechanisms. Ang II increased LC3 I and total LC3 (LC3 I + LC3 II) protein levels in VSMC and RASMC. These results suggest that Ang II stimulates LC3 I processing to LC3 II and also regulates total LC3 content, independent of the autophagy induction elicited by this peptide. We have found discrepancies between RASMC and A7r5 cells. The observation that increased LC3 II protein levels in response to Ang II is unaffected by cycloheximide and actinomycin D may suggest that A7r5 cells may have higher baseline autophagic flux or are more susceptible to stress after the administration of these inhibitors.

Our previous study also showed that Ang II increased total LC3 via an AT1R/ROCK‐dependent mechanism.^7^ The mechanism involves regulating the transcription and translation MAP1LC3B mRNA and an increase in proteolysis and conjugation of LC3. In this context, Derosa et al. showed that higher levels of Bag3 were observed in hypertensive patients compared with healthy subjects.^10^ Furthermore, this study also suggests that regulating Bag3 levels may have a role in cardiometabolic diseases.^10^ This potential link is directly relevant to our study, given that plasma levels of Ang II are increased in hypertension.^10^ In a previous work, we described that Bag3 can regulate LC3 total levels by modulating its mRNA translation. However, Bag3 has no effects on LC3 lipidation.^6^ Our findings suggest that Ang II not only increases total LC3 levels, which may be explained by the increase of Bag3 levels but can also increase LC3 I and LC3 II protein levels, suggesting that Ang II also regulates LC3 post‐translational modifications. Nevertheless, we have not evaluated whether silencing of Bag 3 may impair the increase of LC3 II levels induced by Ang II, which is a limitation of our study. These results suggest that Ang II has a global effect on LC3 regulation, an essential protein in the autophagic process.

## CONFLICT OF INTEREST

The authors confirm that there are no conflicts of interest.

## AUTHOR CONTRIBUTIONS

**David Mondaca‐Ruff:** Conceptualization (equal); Formal analysis (equal); Investigation (equal); Methodology (equal); Writing – original draft (equal); Writing – review & editing (equal). **Clara Quiroga:** Formal analysis (equal); Methodology (equal); Supervision (equal); Writing – review & editing (equal). **Jaime Riquelme:** Formal analysis (equal); Validation (equal); Visualization (equal); Writing – original draft (equal); Writing – review & editing (equal). **Alejandra San Martín:** Funding acquisition (equal); Methodology (equal); Resources (equal); Supervision (equal); Validation (equal); Writing – review & editing (equal). **Mario Bustamante:** Formal analysis (equal); Investigation (equal); Validation (equal); Visualization (equal). **Sergio Lavandero:** Funding acquisition (equal); Resources (equal); Supervision (equal); Validation (equal); Writing – review & editing (equal). **Mario Chiong:** Conceptualization (lead); Funding acquisition (equal); Project administration (equal); Resources (equal); Supervision (equal); Validation (equal); Visualization (equal); Writing – original draft (equal); Writing – review & editing (equal). **Ignacio Norambuena‐Soto:** Formal analysis (equal); Funding acquisition (equal); Investigation (equal); Writing – original draft (equal).

## Data Availability

Data available on request from the authors.
